# Functional Modules in Gametogenesis

**DOI:** 10.3389/fcell.2022.914570

**Published:** 2022-05-26

**Authors:** Mariko Kikuchi, Minoru Tanaka

**Affiliations:** Laboratory of Reproductive Biology, Graduate School of Science, Nagoya University, Nagoya, Japan

**Keywords:** gametogenesis, germ cell, sex, meiosis, folliculogenesis

## Abstract

Gametogenesis, the production of eggs and sperm, is a fundamental process in sexually reproducing animals. Following gametogenesis commitment and sexual fate decision, germ cells undergo several developmental processes to halve their genomic size and acquire sex-specific characteristics of gametes, including cellular size, motility, and cell polarity. However, it remains unclear how different gametogenesis processes are initially integrated. With the advantages of the teleost fish medaka (*Oryzias latipes*), in which germline stem cells continuously produce eggs and sperm in mature gonads and a sexual switch gene in germ cells is identified, we found that distinct pathways initiate gametogenesis cooperatively after commitment to gametogenesis. This evokes the concept of functional modules, in which functionally interlocked genes are grouped to yield distinct gamete characteristics. The various combinations of modules may allow us to explain the evolution of diverse reproductive systems, such as parthenogenesis and hermaphroditism.

## Introduction

For sexually reproductive organisms, life begins with the fusion of two haploid gametes, an egg and a sperm. Gametes are generated from common germline stem cells through tightly regulated developmental processes. Once germline stem cells undergo gametogenesis commitment and sperm-egg fate decisions, the cells halve their genomic size through meiosis. Germline stem cells go through folliculogenesis or spermatogenesis to acquire sex-specific characteristics such as cellular size, motility, and polarity. Although the mechanisms underlying the development of eggs and sperm have been well studied, the initial integration of meiosis, sexually dimorphic folliculogenesis, and spermatogenesis remains elusive.

In mice, the first sexual difference in germ cell development is indicated by the timing of meiosis initiation. Ovarian germ cells initiate meiosis before birth, whereas testicular germ cells do not embark on meiosis until puberty (Spiller and Bowles, 2019). Several signaling pathways involved in meiotic initiation have been identified. One is retinoic acid (RA) signaling, which activates the downstream transcription factor *Stra8* (*stimulated by retinoic acid 8*) and *Meiosin* (*meiosis initiator*) to drive meiotic gene expression (Bowles et al., 2006; Koubova et al., 2006; Ishiguro et al., 2020). Another is BMP (bone morphogenetic protein) signaling, which is essential for meiosis initiation *in vivo* and *in vitro*, and ligands are presumably secreted from ovarian somatic cells (Wu et al., 2016; Miyauchi et al., 2017; Nagaoka et al., 2020). The other is WNT/*β*-catenin signaling, which is required for *Stra8* expression in germ cells, although whether this signaling acts on germ cells directly or indirectly remains unclear (Tomizuka et al., 2008; Naillat et al., 2010; Chassot et al., 2011). In contrast, in the testes, RA is degraded by Cyp26b1 (cytochrome P450 family 26 subfamily B member 1) expressed in somatic cells, thereby preventing germ cells from entering meiosis during embryogenesis (Bowles et al., 2006; Koubova et al., 2006). Additionally, male-specific *Fgf9* (*fibroblast growth factor 9*) also contributes to meiosis suppression *via* the upregulation of *Nanos2* (*nanos C2HC-type zinc finger 2*) in germ cells, thereby making them less responsive to RA (Suzuki and Saga, 2008; Bowles et al., 2010). Instead, germ cells enter mitotic arrest and are specified as spermatogonial cells (Spiller and Bowles, 2019).

Although the molecular mechanisms that promote or suppress meiosis have been investigated intensively in mice, it is difficult to identify germ cell sex determinants hidden among intertwined developmental processes. Meiosis initiation *per se* is not a female-specific event; thus, it is not equivalent to germline feminization. The absence of mouse ovarian germline stem cells also makes the sexual fate decision of the germline difficult to be clarified (Tanaka, 2016). In male mice, certain genes, including *Nanos2*, *Piwil4* (*piwi like RNA-mediated gene silencing 4*), and *Dnmt3l* (*DNA methyltransferase 3 like*), are specifically induced when germ cells are fated to undergo spermatogenesis (Tsuda et al., 2003; La Salle et al., 2004; Aravin et al., 2008). However, in some teleost species, *nanos2* is expressed not only in spermatogonia, but also in oogonia (Aoki et al., 2009; Nakamura et al., 2010; Beer and Draper, 2013), suggesting that a gene(s) induced in male germ cells can be recognized as the acquisition of stemness rather than germline masculinization.

The teleost fish medaka (*Oryzias latipes*) is one of the ideal vertebrate models, in which the presence of germline stem cells in both ovaries and testes has been proven experimentally (Nakamura et al., 2010). This makes it possible to analyze the initial step of sexual development by comparing the profiles of ovarian and testicular germline stem cells. Consequently, we are beginning to understand the presence of a modular structure in gametogenesis that yields sex- and gamete-specific characteristics. This view may explain the diversity observed among reproductive systems.

## Germ Cell Proliferation in Early Gametogenesis

Germline stem cells produce progenitor cells that proliferate mitotically and enter meiosis (Wu et al., 2013). In diverse organisms, germline stem cells undergo two types of mitotic cell division. One is stem-type cell division that produces isolated daughter cells. The other is transit-amplifying cell division, in which several rounds of cell division followed by incomplete cytokinesis form a cluster of cells that are connected *via* intercellular bridges (Pepling et al., 1999; Spradling et al., 2011).

In medaka, stem-type and transit-amplifying cell divisions are referred to as type-I and type-II cell divisions, respectively (Saito et al., 2007; Nakamura et al., 2010; Sumita et al., 2021). Type I/II cell division can be observed in adult testes and ovaries, supporting the presence of germline stem cells in both sexes (Nakamura et al., 2010; Sumita et al., 2021). Importantly, medaka germ cells transplanted into gonads of the other sex give rise to gametes according to the recipient sex, suggesting that germline stem cells are sexually undifferentiated (Tanaka, 2016). In other words, medaka germline stem cells continuously make sexual fate decisions at the time of their commitment to gametogenesis throughout their reproductive life. This prompted us to explore the germ cell sex determinants acting on gametogenesis-committed germ cell types.

Of note, *stra8* has been lost from the medaka genome (Pasquier et al., 2016). In addition, medaka germ cells are not responsive to RA signaling at the time of sex determination (Adolfi et al., 2016; Adolfi et al., 2021). Therefore, factors other than RA signaling components are involved in germline sex determination in medaka.

## Role of Foxl3 in Germ Cell Sex Determination in Medaka

By comparing the transcriptomes of medaka germ cells between genetic males (XY) and females (XX), a switch gene for the sexual fate decision of germ cells, *foxl3* (*forkhead box L3*), has been identified (Nishimura et al., 2015). Foxl3 is initially expressed in type I germ cells in both sexes, but its expression is maintained only in female type II germ cells (Nishimura et al., 2015; Nishimura and Tanaka, 2016). A loss-of-function mutant of *foxl3* leads to germ cell-specific sex reversal in XX gonads and spermatogenesis in the ovarian environment (Nishimura et al., 2015). In addition, transplantation of *foxl3* ^−/−^ germ cells into wild-type ovaries results in spermatogenesis of *foxl3* ^−/−^ germ cells, showing that Foxl3 functions in a cell-autonomous manner as a germ cell sex determinant in medaka ovaries (Nishimura et al., 2015).

Foxl3 is widely conserved in vertebrate genomes except for placental mammals (Bertho et al., 2016). Although the role of Foxl3 in other vertebrate species remains largely unknown, its expression in germ cells has been reported in some teleost species including Nile tilapia (*Oreochromis niloticus*) and Japanese eel (*Anguilla Japonica*) (Wu et al., 2019; Dai et al., 2021). Furthermore, a loss-of-function mutant of tilapia *foxl3* leads to production of spermatogenic cells in XX ovaries (Dai et al., 2021), suggesting a conserved role of *foxl3* in germ cell feminization.

Dmrt1 may play an important role in germline sexual development by antagonizing *foxl3* function. In tilapia, loss of *foxl3* leads to *dmrt1* expression in XX gonads, whereas *dmrt1* mutation causes ectopic *foxl3* expression in XY gonads. This suggests that sexual fate of tilapia germline is regulated by antagonistic roles of *foxl3* and *dmrt1* (Dai et al., 2021). Another important player in germline sexual development is germ cell itself. In medaka, germ cells have an inherent feminizing effect during early gonadal development, which acts independently of developmental stage and sex of germ cells (Kurokawa et al., 2007; Morinaga et al., 2007; Nakamura et al., 2012; Nishimura et al., 2018). The same effect of germ cells has been reported in zebrafish (Slanchev et al., 2005; Tzung et al., 2015), where germ cell apoptosis triggers testis differentiation from juvenile ovaries (Uchida et al., 2002). This could be explained by two juxtacrine signaling pathways. One secreted from germ cells suppresses gonadal masculinization *via* *dmrt1* downregulation, while the *dmrt1*-downstream signal secreted from somatic cells suppresses germline feminization *via* *foxl3* downregulation in germ cells.

Although a full view of the Foxl3 regulatory network remains elusive, RNA sequencing has revealed genes downstream of *foxl3* (Kikuchi et al., 2019). Comparative transcriptome analysis between wild-type and *foxl3* ^−/−^ XX germ cells identified 1,480 differentially expressed genes (DEGs), comprising 600 upregulated and 880 downregulated genes. Gene ontology and pathway enrichment analyses suggest that, in female germ cells, pathways related to the extracellular matrix, oogenesis, and RNA regulation are activated, while the cytoskeletal network and cell cycle pathway are suppressed. These results indicate that Foxl3 may promote germ cell feminization *via* the transcriptional activation of oogenesis-related genes and reorganization of the microtubule network.

In addition, discovery of the Foxl3-binding motif (5′-DHAAACAA-3′) and *in silico* searches for the motif within DEG promoter regions suggest that Foxl3 may bind to most DEG promoters and directly regulate their expression (Kikuchi et al., 2019). Foxl3 likely initiates regulatory events as a pioneer transcription factor that determines the sexual fate of germ cells by targeting silent chromatin and enabling states of its competence to be activated or repressed (Zaret and Mango, 2016). Many pioneer factors are involved in cell fate decisions, as exemplified by FoxA, which persists at enhancer elements in chromatin as endoderm is induced to liver fate (Gualdi et al., 1996; Bossard and Zaret, 2000; Lee et al., 2005).

## Foxl3 Initiates Distinct Genetic Pathways Regulating Meiosis and Folliculogenesis

The following question arises: Which developmental processes does Foxl3 initiate to yield egg characteristics? Recent studies have revealed two genes, *rec8a* (*REC8 meiotic recombination protein a*) and *fbxo47* (*F-box protein 47*) to be direct targets of Foxl3. Similar to *foxl3*, *rec8a* and *fbxo47* are expressed in female type II germ cells, and loss-of-function mutations cause female-specific sterility and ovarian dysgenesis, suggesting their crucial roles in oogenesis (Kikuchi et al., 2019; Kikuchi et al., 2020).

Medaka *rec8a* is one of two mammalian *Rec8* orthologs originating from a teleost-specific whole-genome duplication event (Braasch et al., 2016). *rec8a* encodes an α-kleisin subunit of the meiotic cohesin. The cohesin complex forms a ring-like structure that holds sister chromatids during mitosis and meiosis. Meiotic cohesin is required not only for sister chromatid cohesion, but also for chromosomal axis formation, association of homologous chromosomes, meiotic recombination, and accurate chromosome segregation (Ishiguro, 2019). Mouse *Rec8*-null mutants of both sexes fail to complete meiotic prophase I and become sterile (Bannister et al., 2004; Xu et al., 2005). Similarly, medaka *rec8a* ^–/–^ fish display defects in synapsis between homologous chromosomes during meiotic prophase I, which results in meiotic arrest at the pachytene-like stage. The phenotypes of medaka *rec8a* ^–/–^ are female specific: medaka *rec8a* ^–/–^ males are fertile and develop normal testes containing mature sperm (Kikuchi et al., 2020). It seems likely that the *rec8a* paralogous gene *rec8b* contributes to spermatogenesis. Nevertheless, *rec8a* was the first gene to be identified as a female-specific factor involved in meiosis and may provide a clue to explore the molecular mechanisms regulating sexual dimorphism in meiosis (Cahoon and Libuda, 2019; Sardell and Kirkpatrick, 2020).

Another Foxl3 target, *fbxo47*, encodes a member of the F-box protein family. The F-box protein is widely conserved in eukaryotes and is involved in various cellular functions (Cardozo and Pagano, 2004). F-box proteins are known to act as components of Skp1/Cullin/F-box-protein (SCF) E3 ubiquitin ligase or independently of the ubiquitin-mediated pathway (Kipreos and Pagano, 2000). The medaka *fbxo47* ^−/−^ mutant mimics the phenotype of *foxl3* ^−/−^: Precocious spermatogenesis in XX ovaries. Moreover, *fbxo47* acts genetically upstream of the folliculogenesis-related transcription factors *lhx8b*, *figla*, and *nobox* (Kikuchi et al., 2020). These results indicate that medaka *fbxo47* is involved in the suppression of spermatogenesis and progression of folliculogenesis.

*Caenorhabditis elegans* FBXO47 (also known as PROM-1) mediates the mitosis-meiosis transition *via* ubiquitination and degradation of cyclin E1 (CYE-1), and SCFPROM-1 also promotes homologous chromosome pairing as a positive regulator of CHK-2 serine/threonine kinase (Mohammad et al., 2018). In addition, a recent study revealed that mouse Fbxo47 prevents precocious disassembly of the synaptonemal complex independently of SCF E3 ligase (Tanno et al., 2022). Thus, Fbxo47 may have conserved roles in gametogenesis *via* ubiquitination-dependent and ubiquitination-independent pathways.

**FIGURE 1:**
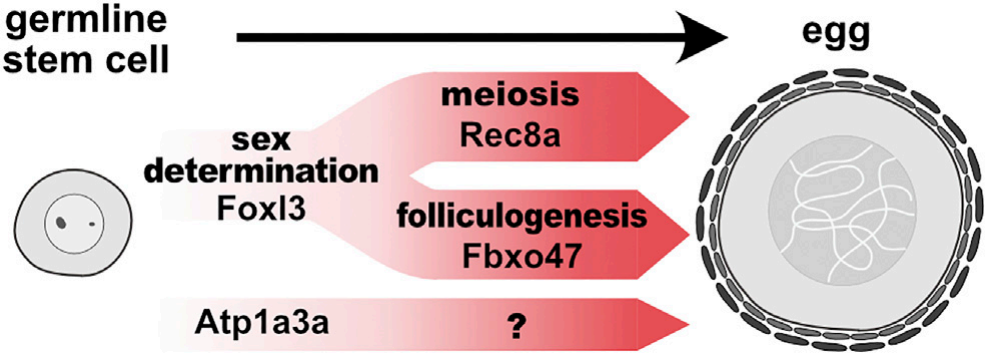
Genetically distinct pathways promote germline feminization in medaka. In germline stem cells, *foxl3*, an intrinsic factor of germ cell sex, directly activates expression of *rec8a* and *fbxo47* to initiate meiosis and folliculogenesis, respectively. Other female-specific pathways (e.g., *atp1a3a*) may also promote another module independently of *foxl3*.

Importantly, epistasis analysis revealed that pathways involving *rec8a* and *fbxo47* are genetically independent (Kikuchi et al., 2020). Hence, the studies described above shed light on two genetic pathways acting downstream of *foxl3*: one promotes female-specific meiosis and the other regulates folliculogenesis and suppression of spermatogenesis (Figure 1). The mechanisms underlying the suppression of spermatogenesis will be an important area for future research.

## Model: Functionally Distinct Modules Integrate Gametogenesis

Accumulating evidence based on genetic analyses indicates that meiosis, folliculogenesis, and spermatogenesis are genetically dissociable. In mice, *Stra8*-deficient ovarian germ cells develop into oocyte-like cells without undergoing meiosis (Dokshin et al., 2013). Loss-of-function mutants of medaka *rec8a* also lead to meiotic arrest of ovarian germ cells at the pachytene-like stage, but they still express transcription factors involved in folliculogenesis, including *lhx8b*, *figla*, and *nobox* (Kikuchi et al., 2020). Meanwhile, medaka *dmc1* ^−/−^ testes can produce a small number of motile sperm with the ability to inseminate, although the ploidy is abnormal (Chen et al., 2016). In contrast, meiosis progresses when folliculogenesis or spermatogenesis is disrupted. *Lhx8* ^−/−^ female mice display severe defects in folliculogenesis, with no abnormalities involving meiotic marker expression and chromosomal structures (Choi et al., 2008). The same phenomenon occurs in medaka *fbxo47* ^−/−^ ovaries, where folliculogenesis is severely disrupted, while the meiotic gene *rec8a* is normally expressed and spermatogenesis proceeds (Kikuchi et al., 2020). Collectively, these phenotypic analyses clearly indicate that although meiosis is dispensable for gametogenesis, processes driving folliculogenesis and spermatogenesis can be activated in the absence of meiosis.

This evokes the concept of ‘functional modules’ where expression of a group of genes are tightly coordinated to act in the same developmental process (Niehrs and Pollet, 1999; Szenker-Ravi et al., 2022), featuring essential gamete characteristics. For example, a module of meiosis contributes to halving the genome, whereas a module of folliculogenesis and spermatogenesis renders gametes feminized or masculinized, respectively.

**FIGURE 2:**
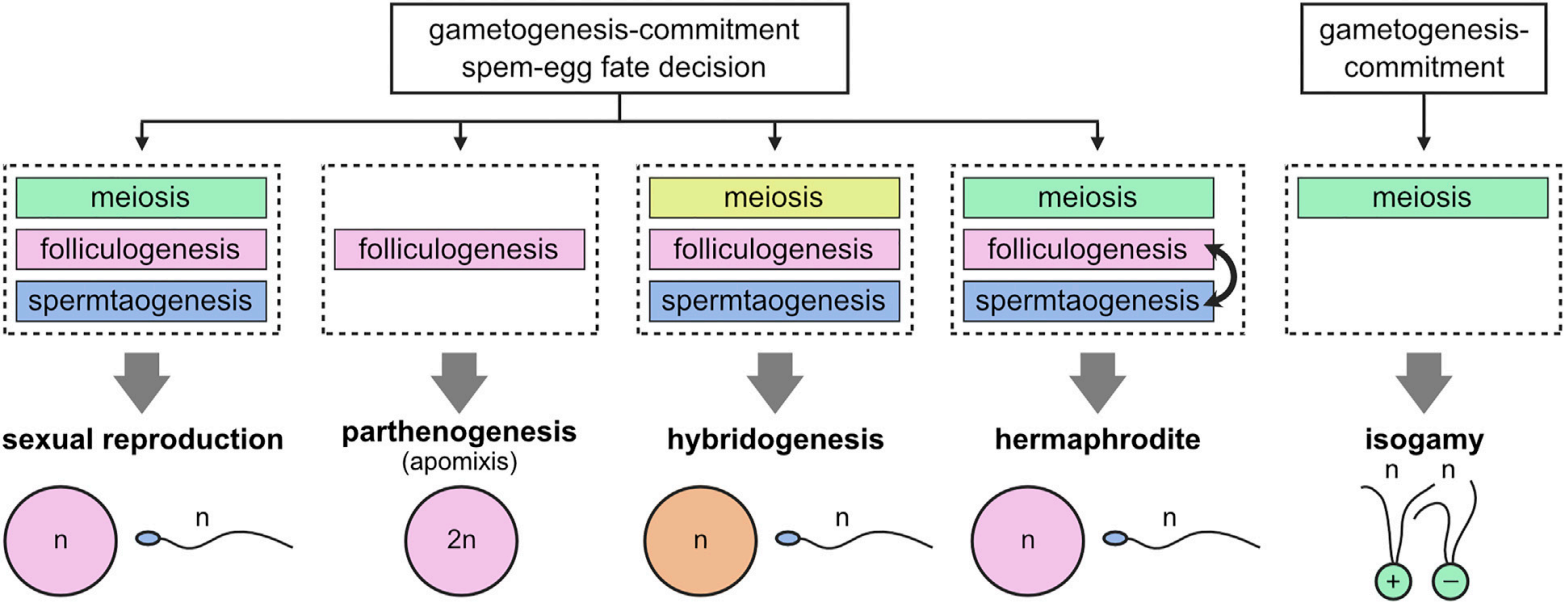
Modular structure of gametogenesis. Initial events of gametogenesis-commitment and sperm-egg fate decision trigger distinct developmental processes, corresponding to distinct modules (meiosis, folliculogenesis, and spermatogenesis). Gain, loss, or alteration of modules during evolution can explain adaptive diversification of reproductive systems.

This raises the interesting view that modules can be referred to as the basis for creating various modes of reproduction during evolution (Figure 2). In apomictic parthenogenesis (development of embryos from unfertilized eggs), bypassing meiotic modules allows gametogenesis to circumvent meiosis, resulting in the production of diploid eggs (Lampert, 2008; Mirzaghaderi and Horandl, 2016). This type of reproduction has been reported in bdelloid rotifers and many arthropods (Simon et al., 2003). Another unisexual mode of reproduction, hybridogenesis, could also be explained by alterations to the meiotic module so that one of the parental genomes is selectively transferred to the gametes, while the other one is lost during meiosis (Lehtonen et al., 2013; Lavanchy and Schwander, 2019). Hybridogenesis has been found in a diverge range of animals, including fishes (*Poeciliopsis lucida-monacha*), frogs (*Pelophylax esculentus*), and insects (*Bacillus rossius-grandii*) (Schultz, 1969; Mantovani and Scali, 1992; Vorburger et al., 2009). Additionally, hermaphrodites (e.g., *Caenorhabditis elegans*) consecutively produce egg and sperm from germline stem cells by acquiring a mechanism for switching a module of folliculogenesis from that of spermatogenesis (Hubbard and Greenstein, 2000). In addition, sexually dimorphic (anisogamous) gametes seem to have descended from equal-sized (isogamous) gametes, which is common in algae and protists (Kirk, 2006). Thus, during the course of evolution, the folliculogenesis and spermatogenesis modules might have been progressively evoked and selected to develop large eggs and small sperm.

Regarding folliculogenesis, it is likely that this module diverges into other submodules, leading to more specific gametogenic characteristics, such as maternal RNA accumulation and fertilization capability. Additionally, the finding of oogenesis-specific, but *foxl3*-independent, gene expression suggests that characteristics independent of meiosis and folliculogenesis are also present. *Atp1a3a* (*ATPase Na+/K+ transporting subunit alpha 3a*) is one of the genes that we found from our RNA-seq data to be upregulated specifically in female germ cells but independently of *foxl3* (Kikuchi et al., 2019) (Figure 1). During oogenesis, this cation-exchanging transporter could be involved in cell enlargement by regulating osmotic pressure.

In conclusion, we propose a modular structure of gametogenesis, in which genetically distinct modules build the development of functional gametes. Modification and/or loss of modules will provide a way to explain the diversification of reproductive systems.

## Data Availability

The original contributions presented in the study are included in the article/Supplementary Material, further inquiries can be directed to the corresponding author.
